# Arsenic mobilization in a high arsenic groundwater revealed by metagenomic and Geochip analyses

**DOI:** 10.1038/s41598-019-49365-w

**Published:** 2019-09-10

**Authors:** Zhou Jiang, Ping Li, Yanhong Wang, Han Liu, Dazhun Wei, Changguo Yuan, Helin Wang

**Affiliations:** 1 State Key Laboratory of Biogeology and Environmental Geology, China University of Geosciences, Wuhan, 430074 P.R. China; 2 School of Environmental Studies, China University of Geosciences, Wuhan, 430074 P.R. China

**Keywords:** Microbial ecology, Element cycles

## Abstract

Microbial metabolisms of arsenic, iron, sulfur, nitrogen and organic matter play important roles in arsenic mobilization in aquifer. In this study, microbial community composition and functional potentials in a high arsenic groundwater were investigated using integrated techniques of RNA- and DNA-based 16S rRNA gene sequencing, metagenomic sequencing and functional gene arrays. 16S rRNA gene sequencing showed the sample was dominated by members of *Proteobacteria* (62.3–75.2%), such as genera of *Simplicispira* (5.7–6.7%), *Pseudomonas* (3.3–5.7%), *Ferribacterium* (1.6–4.4%), *Solimonas* (1.8–3.2%), *Geobacter* (0.8–2.2%) and *Sediminibacterium* (0.6–2.4%). Functional potential analyses indicated that organics degradation, assimilatory sulfate reduction, As-resistant pathway, iron reduction, ammonification, nitrogen fixation, denitrification and dissimilatory nitrate reduction to ammonia were prevalent. The composition and function of microbial community and reconstructed genome bins suggest that high level of arsenite in the groundwater may be attributed to arsenate release from iron oxides reductive dissolution by the iron-reducing bacteria, and subsequent arsenate reduction by ammonia-producing bacteria featuring *ars* operon. This study highlights the relationship between biogeochemical cycling of arsenic and nitrogen in groundwater, which potentially occur in other aquifers with high levels of ammonia and arsenic.

## Introduction

Arsenic (As) is a toxic metalloid and is widespread in the environment. Elevated As in groundwater endangers the health of hundreds of millions of people worldwide, especially in Southern and Southeastern Asia^1,2^.The widespread problem of high levels of As in groundwaters prompts intensive studies from multi-disciplinary research fields, such as geochemistry, hydrology, mineralogy, microbiology and ecology, to understand the hydro-biogeochemical behaviors of As in global aquifer systems^3^. To date, three main mechanisms have been proposed for As mobilization in groundwater: (1) the reductive dissolution of As-bearing iron (Fe) oxides under reducing conditions^4,5^, (2) the oxidative dissolution of As-bearing pyrite and arsenopyrite under oxidizing conditions^6,7^, and (3) the competitive adsorption between As and phosphate/bicarbonate under alkaline conditions^8,9^.

The first mechanism commonly prevails in the late Pleistocene-Holocene aquifer systems with the sedimentation of fresh organic matter, such as inland basins from West Bengal, Bangladesh, Nepal, Cambodia, Vietnam and China, where degradation of organic matter fuels microorganisms to reduce and dissolve As-bearing iron oxides^4,10,11^. Among those microbes, iron-reducing and sulfate-reducing bacteria are known to play important roles in As mobilization. For example, dissimilatory iron-reducing bacteria, such as *Geobacter* sp. and *Shewanella* sp., could directly utilize organic carbon as electron sources to reduce As-bearing iron oxides, leading to As release or re-immobilization by forming secondary iron minerals, such as vivianite and magnetite^12–14^. Sulfide generated by sulfate-reducing bacteria in aquifers abiotically reduces As-bearing iron oxides to mobilize As^15–17^. If derived ferrous iron is sufficiently available in groundwater, sulfide could form iron-sulfide minerals to further sequester As through co-precipitation and/or adsorption^18–20^. However, the reductive dissolution mechanism only accounts for the initial trigger of arsenate release from iron oxides, but not for the transformation from arsenate to arsenite in reducing aquifers. Previous studies based on *arrA* gene coding respiratory arsenate reductase large subunit highlight the role of dissimilatory arsenate-respiring bacteria on arsenate transformation^13,21,22^. In contrast to sporadic arsenate respiring organisms, As detoxifying organisms harboring arsenic-resistance (*ars*) operon are much more prevalent among prokaryotes and well known to contribute to arsenate reduction^23–25^. In addition, it was reported that microbial oxidation and methylation of arsenite occur in some groundwater^26,27^. Therefore, to comprehensively understand As transformation processes in groundwater systems, those functional genes of As reduction, oxidation and methylation should be evaluated together using some high-throughput molecular technologies, such as metagenome and Geochip^28^. Besides, the integrated metabolic potential of metagenome and Geochip enables As metabolism to link to other corresponding elements cycle, such as C, N and S, and further elucidate their relationship and role in As mobilization. With the complementary beneficial characteristics including new genes discovery, low detection limits, high reproducibility, and potential for quantification, metagenome and Geochip could be combined to reveal microbial functional potentials in specific ecosystems of interest.

High levels of As in groundwater systems is often accompanied by high ammonium concentrations, such as Hetao basin, China^29^, Bengal delta plain and Ganges River floodplain, Bangladesh^30,31^, Red River floodplain, Vietnam^32^, and Mekong Delta, Cambodia^33^. It was reported that ammonium in groundwaters reflects degradation of organic matter and/or inputs of anthropogenic activities, such as fertilizers and manure, and only act on the formation of reducing conditions to favor the reductive dissolution of As-bearing Fe oxides^32,34,35^. Recently, isotopic evidence of nitrogen sources in As-contaminated groundwater suggested the prevalence of nitrification, denitrification, ammonification and dissimilatory nitrate reduction to ammonia (DNRA)^34,36,37^. These results infer that some nitrogen metabolic processes possibly link to As mobilization in groundwater systems. After all, none of the above iron-reducing, sulfate-reducing and arsenate-reducing bacteria could reasonably explain the positive correlation between As and ammonium concentrations in groundwater.

The objective of this study is therefore to further understand the microbial mobilization and transformation processes of As in groundwater, especially the role of nitrogen metabolism on As mobilization. To this end, we used the integrated techniques of RNA- and DNA-based 16S rRNA gene sequencing, metagenomic sequencing and functional gene array (Geochip 4.0) to assess microbial community composition and functional potential in a high As groundwater. This groundwater is located in Hetao Basin, Inner Mongolia, China, a representative endemic arsenicosis area with an affected population of more than two hundred thousand^38^. Our results indicated that some ammonium-producing bacteria with *ars* operon is likely to mediate the transformation from arsenate to arsenite in groundwater after it is released from the reductive dissolution of As-bearing iron oxides by iron-reducing bacteria.

## Material and Methods

### Field sampling

The hand-pumped tube well (N40.96683°, E107.00517°) from resident Dingshan Li in Shahai village, Hangjinhouqi County, western Hetao Basin, was selected for this study because of high levels of As detected and arseniasis family history (two members died of long-term drinking groundwater). The sample herein was abbreviated LDS. Groundwater biomass were collected using both normal filtration, with a 0.22 μm × 142 mm (pore size × diameter) nitrocellulose membrane filter (Merck Millipore, Billerica, MA, USA), and tangential flow filtration (TFF; Prep/Scale Spiral Cartridge with 30 kDa molecular weight cutoff, Millipore, Billerica, MA, USA)^39^. The filtered water was collected into 100 mL acid-washed polypropylene bottle (to measure As species), brown glass bottle (to measure dissolved organic carbon, DOC) and serum vial with butyl rubber septum stoppered and aluminum seal (to measure methane). The filtered membrane was cut into two equal parts and placed into 15 mL sterile polypropylene tubes with/without prefilled Ambion RNA *later* Solution (ThermoFisher Scientific, Waltham, WA, USA) (RNA and DNA extraction for 16S rRNA gene sequencing) respectively. Concentrated cell suspension by TFF was packed into 500 mL sterile nuclease-free plastic bottles (DNA extraction for metagenomic sequencing and Geochip analyses). The groundwater volumes of normal filtration and TFF is approximately 10 L and 2000 L respectively. All samples were transported to the laboratory on dry ice and then stored at −80 °C until further analyses.

### Geochemical measurements

Geochemical parameters including temperature, pH, electrical conductivity and oxidation-reduction potential (ORP) were measured *in situ* using a portable multi meter (HQ40D, Hach, Loveland, CO, USA). Concentrations of sulfide, sulfate, ammonium, nitrite, nitrate, phosphate, ferrous iron and total iron in groundwater were determined at the sampling with a portable colorimeter (DR890, Hach, Loveland, CO, USA) according to the manufacture’s procedures. In the laboratory, four As species of arsenite, arsenate, monomethylarsenate (MMA) and dimethylarsonate (DMA) were performed using liquid chromatography combined to hydride generation atomic fluorescence spectrometry (AFS-9780, Haiguang, Beijing, China). Dissolved organic carbon (DOC) concentrations were determined using a TOC analyzer (Vario MICRO cube, Elementar, Langenselbold, Germany). Soluble methane concentrations were analyzed by gas chromatography (Thermo Trace Ultra) at the Third Institute of Oceanography, State Oceanic Administration of China. All samples were run in triplicate and then averaged.

### DNA and RNA extraction and cDNA synthesis

Cells in TFF concentrates were pelleted by centrifugation at 6000 rcf for 10 min after defrosting. Genomic DNA was extracted from the pellet and filter using MoBio PowerSoil DNA Isolation Kit (Qiagen, Valencia, CA, USA) according to manufacturer’s instructions. Total RNA was extracted from the filter preserved in Ambion RNA*later* Solution using RNeasy Mini Kit (Qiagen, Valencia, CA, USA). An Ambion Tubro DNA-free Kit (ThermoFisher Scientific, Waltham, WA, USA) was used to digest residual DNA. To verify complete removal of genomic DNA in total RNA, we utilized total RNA as a template to amplify 16S rRNA gene with general primer 515F (5′-GTGCCAGCMGCCGCGGTAA-3′) and 806R (5′-GGACTACHVGGGTWTCTAAT-3′) and performed electrophoresis detection. DNA and RNA concentrations were measured by NanoDrop One (Thermo Fisher Scientific, Waltham, MA, USA).

Reverse transcription-PCR (RT-PCR) amplification of 16S rRNA was achieved using Invitrogen SuperScript III One-Step RT-PCR System with Platinum Taq DNA Polymerase (ThermoFisher Scientific, Waltham, WA, USA). Specifically, it was carried out in a 50 μL reaction containing 25 μL 2 × Reaction buffer, 0.2 μM of both sense and anti-sense primers (515F and 806R), 10–15 ng template and 2 μL SuperScript III RT/Platinum Taq Mix using the following program: cDNA synthesis at 55 °C for 30 min; initial denaturation at 94 °C for 3 min; 35 cycles of 94 °C for 45 s, 50 °C for 60 s, and 72 °C for 90 s; final extension at 72 °C for 10 min. The synthesized cDNA was purified with the QIAquick PCR Purification Kit (Qiagen, Valencia, CA, USA).

### 16S rRNA gene amplification, sequencing and preprocessing

The V4 region of 16S rRNA gene was amplified from both DNA and synthesized cDNA using the primer 515F and 806R combined with Illumina adapter sequences, a pad and a linker, as well as barcodes on the reverse primers. PCR amplification was carried out in a 25 μL reaction buffer containing 12.5 μL GoTaq colorless Master Mix (Promega, Madison, WI, USA), 0.5 μM of both forward and reverse primers, 10–15 ng template using the following program: initial denaturation at 94 °C for 3 min, followed by 35 cycles of 94 °C for 45 s, 50 °C for 60 s, and 72 °C for 90 s, and then a final extension at 72 °C for 10 min. Reactions were performed in triplicate and pooled.

Amplicons were verified by agarose gel electrophoresis and quantified with PicoGreen using a FLUOstar Optima microplate reader (BMG Labtech, Jena, Thuringia, Germany). 500 ng of amplicon from each sample were combined together and purified using MoBio UltraClean PCR Clean-Up Kit (Qiagen, Valencia, CA, USA) and then re-quantified with PicoGreen. Sample library was prepared according to the MiSeq Reagent Kit Preparation Guide (Illumina, San Diego, CA, USA). Briefly, the sample was denatured by mixing 10 μL 2 nM combined amplicon and 10 μL 0.2 mol NaOH and incubated for 5 min at room temperature. Denatured sample was diluted to 7 pM using HT1 buffer and mixed with 7 pM PhiX control (final PhiX control concentration, 10%). A total of 600 μL sample mixture, together with customized sequencing primers for forward, reverse, and index reads, were loaded into the corresponding wells on the cartridge of a 300-cycle MiSeq Reagent Kit v2 and run on an Illumina MiSeq system (Illumina, San Diego, CA, USA). RNA- and DNA-based 16S rRNA gene sequencing raw data were deposited to the Short Read Archive database at NCBI (Accession number: PRJNA493661).

Raw sequencing data with perfect matches to barcodes were split to sample libraries. Forward and reverse reads with at least 30 bp overlap and less than 25% mismatches were joined using Fast Length Adjustment of SHort reads (FLASH)^40^. The joined sequences were trimmed using Btrim with a QC threshold of greater than 30 over a 5 bp window size and a minimum length of 140 bp^41^. After deleting sequence containing N base and trimming by sequence length of <251 and >255 bp, the sequences were clustered to operational taxonomic units (OTUs) by Uparse at a similarity level of 97%^42^. Singletons in generated OTU tables were removed for downstream analyses. The Ribosomal Database Project (RDP) classifier was used to assign the taxonomy of representative sequences at a minimal 50% confidence^43^. Samples were rarefied at 55841 sequences per sample. The above steps were performed through a Galaxy-based pipeline compiled by Ye Deng’s lab in Research Center for Eco-Environmental Sciences, China Academy of Sciences, China (http://mem.rcees.ac.cn:8080).

### Metagenomic sequencing, preprocessing and genome binning

DNA was randomly sheared into small pieces of ~350 bp using Covaris M220 Focused-ultrasonicator (ThermoFisher Scientific, Waltham, WA, USA). Sample library was prepared according to the instruction manual of NEB Next Ultra DNA Library Prep Kit for Illumina (New England Biolabs, Ipswich, MA, USA). Briefly, it consists of fragmented DNA end-repaired, adaptor ligation, size selection and cleanup of adaptor-ligated DNA, PCR enrichment of adaptor-ligated DNA (5 cycles) and cleanup of PCR reaction. The library concentration was determined using Qubit 3.0 Fluorometer (ThermoFisher Scientific, Waltham, WA, USA) and sequenced on an Illumina HiSeq X Ten platform at Guangdong Magigene Biotechnology Co. Ltd (Guangdong, China). ~13 Gbp (2 × 150 bp) reads were generated. Metagenomic sequencing raw data were deposited to the Short Read Archive database at NCBI (Accession number: PRJNA493663).

The raw sequencing data was trimmed to produce clean data using Trimmomatic (Version 0.36)^44^ and *de novo* assembled using MEGAHIT(Version 1.0.6)^45^. The assembled scaffolds were interrupted from N base site and the <500 bp scafftigs were removed. MetaGeneMark(Version 3.38) was used with default parameters to predict the open reading frame (ORF) of scafftigs and those ORFs with a length of <90 bp were omitted^46^. Redundant gene catalogue(Unigenes) was obtained using CD-HIT (Version:4.7) by grouping sequences with an identity of >95% and coverage of >90% into clusters and assigning the longest sequence as representative of the cluster^47^. The clean data was aligned to the Unigenes using BBMap (http://jgi.doe.gov/data-and-tools/bbtools/) to obtain the number of mapped reads of each gene^48^. Based on the number of mapped reads and length of gene, each gene abundance was computed and utilized for subsequent statistical analysis. DIAMOND software was used to blast the Unigenes to the sequences of bacteria, fungi, archaea and viruses extracted from NCBI non-redundant (NCBI-nr) database with the e value threshold of 1e-10^49^. The best hit was chosen to be the taxonomic unit of Unigenes based on the results of lowest common ancestor (LCA) algorithm compiled in MEGAN software^50^. Functional annotation was performed by blasting Unigenes to the functional database including NCBI-nr database, KEGG database (http://www.kegg.jp/kegg/) and eggNOG database (http://eggnogdb.embl.de/) using DIAMOND software with the e value threshold of 1e-10. For each gene’s blast result, the best hit with lowest e value and Blast coverage ratio between query and reference sequences of >40 was chosen for subsequent analysis. In addition, the coverage information of the scaffolds with lengths of >5 kb and tetranucleotide frequency (TNF) were used to perform the genome binning by using MetaBAT with a parameter of verysensitive^51^. The completeness, contamination and strain heterogeneity of genomic bins were then estimated by using CheckM^52^. Finally, three genome bins belonging to *Pseudomonas* and *Acidovorax* were retained for further analysis.

### Geochip 4.0 analysis

The GeoChip 4.0, containing 82074 oligonucleotide probes targeting 141995 genes in 410 gene families involving various element cycle, was used to analyze the functional potential of the sample. The experimental procedures referred to Tu *et al*.^53^ for details. Briefly, the purified DNA was labeled with Cy-3 using random primers and the Klenow fragment of DNA polymerase I and then mixed with sample tracking control (NimbleGen, Madison, WI, USA). With an addition of hybridization buffer, the sample was hybridized at 42 °C for approximately 16 h on a MAUI hybridization station (BioMicro, Salt Lake City, UT, USA). After the hybridization, the array was scanned with a NimbleGen MS200 Microarray Scanner (Roche NimbleGen, Madison, WI, USA) at full laser power. Signal intensities were measured based on scanned images. Poor quality spots with a signal-to-noise ratio of less than 2.0 were removed and the positive signals were normalized before statistical analysis. The sample was run in triplicate and then averaged. GeoChip 4.0 hybridization data process was performed through a pipeline compiled by the Institute for Environmental Genomics, University of Oklahoma, USA (http://ieg.ou.edu/microarray).

## Results and Discussion

### Geochemistry

Sample LDS is under a slightly alkaline reducing condition with a total As concentration of 1.28 mg/L (Table S1). No MMA and DMA were detected and the ratio of arsenite to total As was 0.77, suggesting that inorganic As reduction prevails. Given high concentrations of DOC (8.58 mg/L) and ferrous iron (0.15 mg/L) and total iron (0.66 mg/L), the reductive dissolution of As-bearing iron oxides seems to contribute to As release in the groundwater, similar to previously reported results^54,55^. Sample LDS has extremely low concentrations of sulfide and sulfate (below detection limit), generally excluding the role of sulfur on As mobilization or sequestration^19,56,57^. High concentrations of ammonium (1.02 mg/L) and nitrate (0.79 mg/L) in sample LDS suggest the presence of some nitrogen metabolic processes, such as denitrification or DNRA^34,36^.

### Taxonomic profiling of microbial community

**Figure 1:**
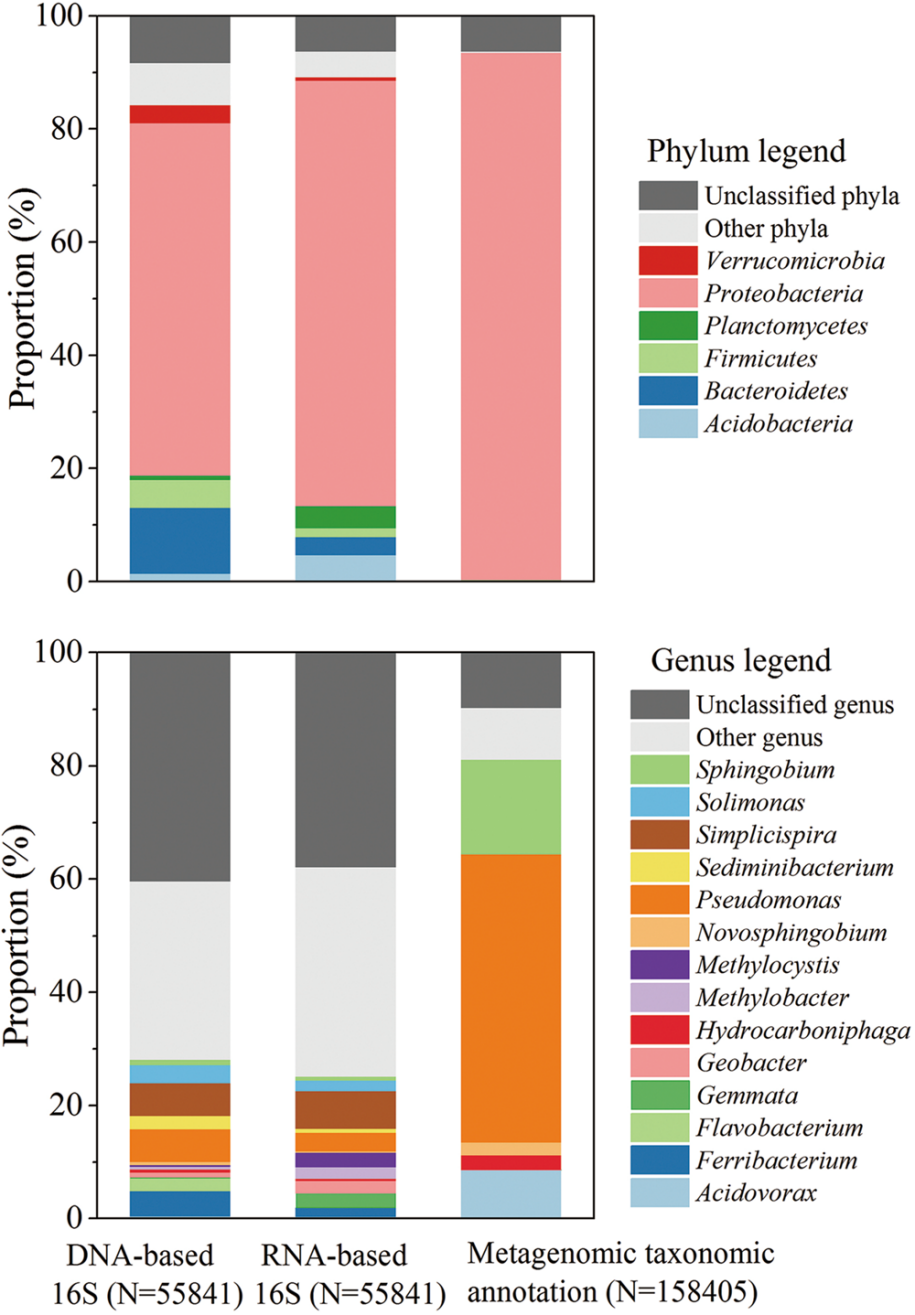
Microbial community composition and structure in sample LDS revealed by RNA- and DNA-based 16S rRNA gene sequencing and metagenomic taxonomic annotation. Upper diagram: at the level of phylum; Down diagram: at the level of genus. Only the phyla and genera with abundances of >2% are displayed. N in parentheses indicates the number of reads (16S rRNA gene) or Unigenes (metagenomic).

The As mobilization and transformation in groundwater systems are mediated by a variety of microorganisms^58^. An integrated analysis of 16S rRNA gene sequencing based on both RNA and DNA and metagenomic taxonomic annotations was used to investigate the composition and structure of microbial community in sample LDS (Figure 1 and Table S2). As the representative of active and present microbial population in sample LDS respectively, RNA- and DNA-based 16S rRNA gene sequencing showed similar results. *Proteobacteria* predominates in sample LDS, with a proportion of 62.3–75.2%. The following phyla are *Bacteroidetes* (3.2–11.6%), *Firmicutes* (1.6–4.9%), *Actinobacteria* (1.4–4.7%), *Planctomycetes* (0.8–3.9%) and *Verrucomicrobia* (0.6–3.2%). At the level of genus, the majority (37.8–40.3%) is unclassified, with the rest consist of *Simplicispira* (5.7–6.7%), *Pseudomonas* (3.3–5.7%), *Ferribacterium* (1.6–4.4%), *Solimonas* (1.8–3.2%), *Sediminibacterium* (0.6–2.4%), *Geobacter* (0.8–2.2%) and numerous rare genera. In contrast to the high sensitivity of 16S rRNA genes, metagenomics sequencing without deep coverage usually detect principal species in complex microbial communities^59,60^. As we found in this study, dominant phylum *Proteobacteria* has a proportion of up to 93.2% in metagenomic taxonomic annotation, in which major genera are mainly composed of *Pseudomonas* (50.9%), *Sphingobium* (16.7%), *Acidovorax* (8.6%), *Hydrogenophaga* (2.6%) and *Novosphingobium* (2.2%).

Typical iron-reducing bacteria detected by 16S rRNA gene sequencing based on both RNA and DNA, such as *Geobacter* and *Ferribacterium*, suggest that iron reduction probably occurs in sample LDS. These iron-reducing bacteria may utilize organic matter as electron donors to reduce As-bearing iron oxides and release absorbed As^5,61,62^. High concentrations of As, iron and DOC detected in sample LDS are consistent with the suggested roles of iron-reducing bacteria (Table S1). Except for reductive dissolution, As-bearing iron oxides might dissociate a small fraction of As into solution due to the competitive adsorption of phosphate in the groundwater (Table S1)^5,8,9^. As the most abundant genus in the sample LDS, *Simplicispira* likely involve nitrate reduction as the strains of the genus *Simplicispira* isolated from freshwater, soil, and activated sludge were reported to be capable of denitrification^63^. Many members of *Pseudomonas* and *Sphingobium* have demonstrated to be able to degrade aromatic compounds, and thus those derived intermediates may further serve as electron donors for themselves or other microorganisms to reduce iron, nitrate and arsenate^64,65^.

### Functional potential of microbial community

**Figure 2:**
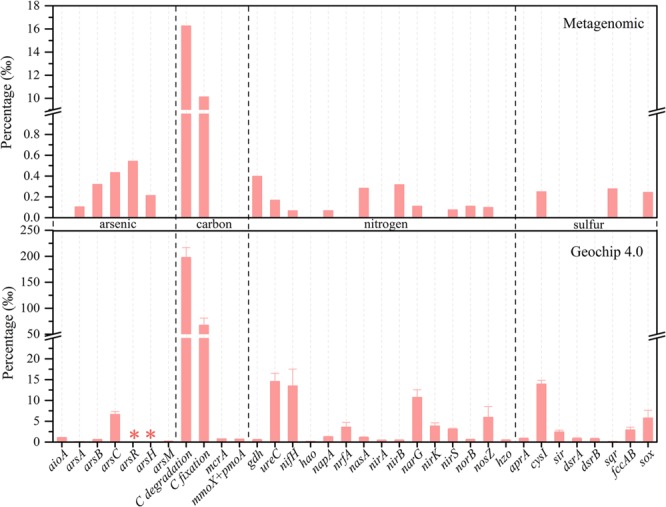
Relative abundances of arsenic, carbon, nitrogen and sulfur-metabolizing genes detected from metagenomic annotations in KEGG database (Upper diagram) and Geochip 4 (Down diagram). The genes with no detection by both metagenomic and Geochip 4, such as *arrA* and *amo*, are not displayed in this figure. Gene abbreviations refer to Table S4 for details. Gene marked by an asterisk represents no corresponding probe and error bar indicates standard deviation among triplicates in Geochip 4.0.

Metagenomic functional annotation and Geochip 4.0 were used to assess microbial functional potentials in sample LDS. Close to 11 Gbp clean data from metagenomic were assembled de novo to scaftigs and further derived 240574 protein-coding genes (Table S3). Among them, 104064 genes were successfully annotated to orthologue numbers in KEGG database. The results from metagenomic and Geochip 4.0 are similar regarding the relative abundances of As, carbon, nitrogen and sulfur-metabolizing genes (Figure 2 and Table S4). High abundances of genes in *ars* cluster (*arsRABCH*) and absence of *arrA* infer that arsenate reduction in sample LDS may be mediated by As-resistant organisms harboring *ars* operon^66^. Arsenite oxidation and methylation are much less prevalent, revealed by near absent *aioA* and *arsM*. These results coincide with high arsenite concentration found and no detectable MMA and DMA in sample LDS. Carbon compounds degradation genes are significantly higher than carbon fixation genes, suggesting the consumption of DOC as energy and electron sources. Detection of *mcrA*, *mmoX* and *pmoA* by Geochip 4.0 indicate that some methanogens and methanotrophs inhabit sample LDS and regulate the methane concentration together (204.8 µg/L) (Table S1). Methanogens detected by sequence- and group-specific probes in Geochip 4.0 are composed of uncultured archaea (60.7%), *Methanoculleus marisnigri* JR1 (10.1%), *Methanocorpusculum labreanum* (8.4%) and *Methanococcus aeolicus Nankai-*3 (6.7%) (Table S5), which is similar to our previous study on *mcrA* libraries in groundwater samples from Hetao basin, Inner Mongolia^67,68^. Regarding nitrogen cycle, the dominant processes in sample LDS mainly include denitrification (presence of a whole set of *narG*, *nirK*, *nirS*, *norB* and *nosZ*), nitrogen fixation (*nifH*) and ammonification (*gdh* and *ureC*). Detections of *napA*, *nrfA*, *nasA* and *nirAB* imply that DNRA and assimilatory nitrate reductions to ammonia may occur. The near absence of *amo*, *hao* and *hzo* suggest inert nitrification and anammox activities. This is also supported by no detection of these characteristic anammox bacteria (genera of *Candidatus Kuenenia*, *Candidatus Brocadia*, *Candidatus Jettenia*, *Candidatus Anammoxoglobus* and *Candidatus Scalindua*) in RNA- and DNA-based 16S rRNA gene sequencing^69^. High levels of ammonium in sample LDS is thus attributed to multiple production pathways of ammonium as well as lack of appreciable ammonium-consuming process. In contrast to low levels of *aprA*, *dsrA* and *dsrB*, high abundances of *cysI* and *sqr* indicate that assimilatory sulfate reduction rather than dissimilatory sulfate reduction dominate in sulfur-deficient sample LDS. The transformation from thiosulfate to sulfate probably occurs, revealed by the presence of *sox*.

Metagenomic and Geochip 4.0 analyses display different results in terms of what microorganism possess above functional genes (Table S5). For example, metagenomic analyses indicate that the majority of *arsC*-harboring bacteria are affiliated with *Pseudomonas*, followed by *Sphingobium*, *Sphingopyxis* and *Acidovorax*. Differently, Geochip 4.0 analyses show the presence of *arsC* gene in *Delftia acidovorans* SPH-1, *Maricaulis maris* MCS10, *Aspergillus fumigatus* Af293, *Shewanella putrefaciens* CN-32, *Shewanella* sp. ANA-3 and *Pseudomonas putida* GB-1. This disparity should be attributed to the different nature of those technologies^70^. It is known that Geochip requires a *priori* sequence information to synthesize probes before hybridization and these probes enable Geochip to identify both abundant and rare species by theirs sensitivity and specificity^70,71^. On the contrary, metagenome without a high sequencing depth only detect those dominant species in complex microbial community, as we found in taxonomic profiling results in this study. Therefore, As-, N- and S-metabolizing functional genes in metagenome are similar to the members from the dominant genera *Pseudomonas*, *Sphingobium* and *Acidovorax*, whereas them in Geochip is more taxonomically diverse (Table S5). It should be noted that the majority of metagenomic ORFs in sample LDS is functionally unknown (Figure S1 and Table S3), suggesting the novelty of sample LDS and highlighting that the relative abundance of functional genes may underestimated in metagenome. Geochip results cannot go beyond the detection capability of the probes fabricated on the array and thus may overestimate relative abundance of corresponding genes. Exactly for those reasons, the relative abundance of functional genes found in metagenomic analysis is much lower than that in Geochip 4.0 (Figure 2).

### Binning of metagenomic

**Figure 3:**
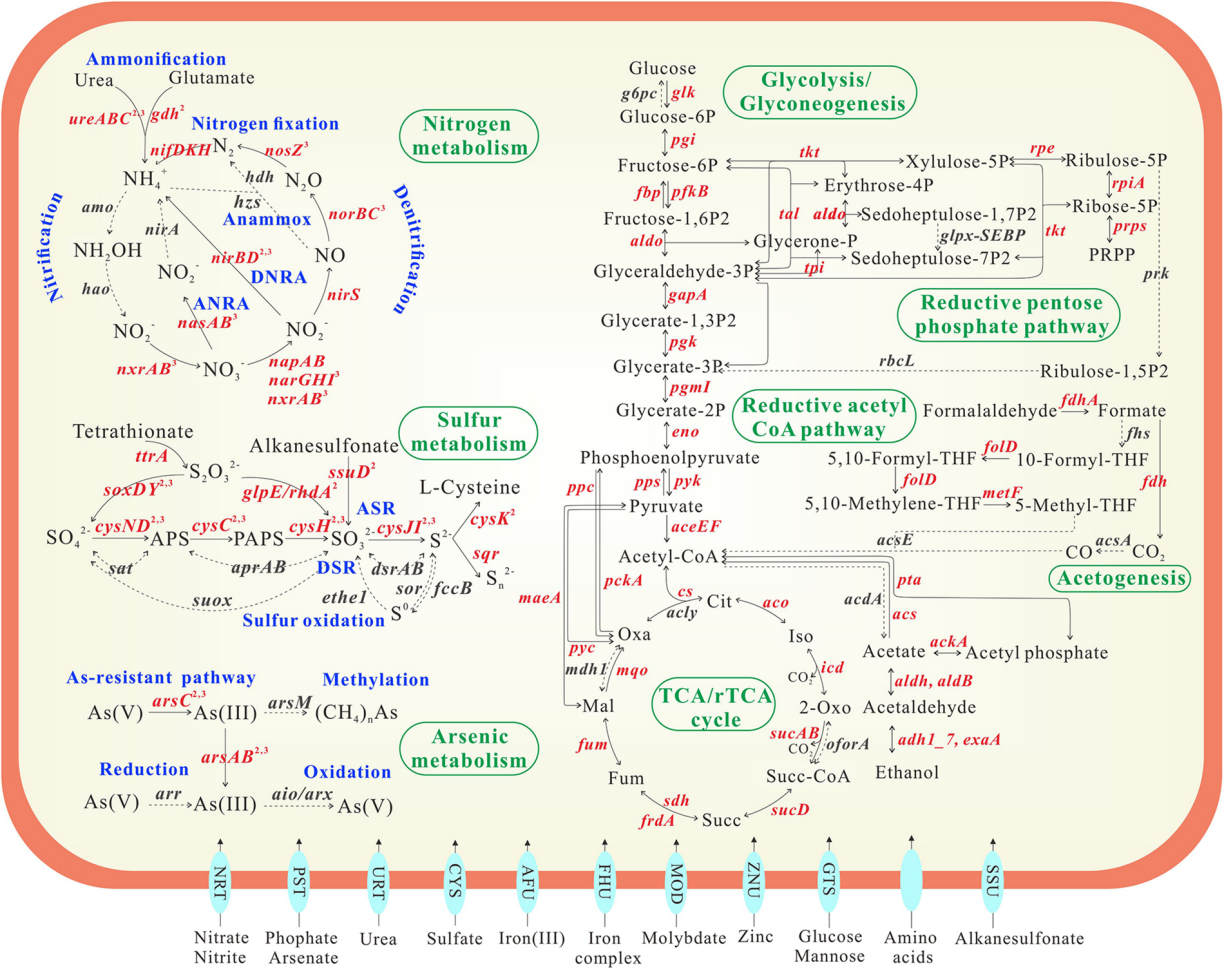
Overview of metabolic potentials in the genome bin 1. Genes related to nitrogen, arsenic, sulfur and carbon metabolisms and membrane transporters are shown. Genes in red font are detected and their corresponding reactions are highlighted by solid arrows. Genes in grey font are not detected and their corresponding reaction are displayed by dotted arrows. Gene abbreviations refer to Table S4 for details. Nitrogen-, arsenic- and sulfur-related genes marked by the superscripts of 2 and 3 indicate their presence in the genome of bin 2 and bin 3. Due to the same detection of genes from carbon metabolism and membrane transporters in all bins, the superscripts of 2 and 3 in these genes are omitted. ANRA: Assimilatory nitrate reduction to ammonia; DNRA: Dissimilatory nitrate reduction to ammonia; ASR: Assimilatory sulfate reduction; DSR: Dissimilatory sulfate reduction.

Three high-quality genomic bins belonging to *Pseudomonas stutzeri*, *Pseudomonas* and *Acidovorax* were derived from metagenomic data in sample LDS (Table S6). CheckM result showed their completeness of 96.5–99.3% and contamination of <1.1%. The genome sizes of bins range from 4.5 Mbp to 5.5 Mbp. The number of protein-coding genes is 4238, 5084 and 5092 respectively. These three bins encode all genes involved in glycolysis and citrate cycle, but lack key genes in reverse citrate cycle (rTCA cycle), reductive acetyl-CoA pathway and reductive pentose phosphate pathway (Table 1 and Figure 3), supporting the idea that these microorganisms utilize organic carbon as energy and electron sources. To cope with As toxicity and sulfur deficiency in sample LDS, they all possess the pathway of As-resistant, assimilatory sulfate reduction and thiosulfate metabolism. Bin 1, *Pseudomonas stutzeri*, participate in other metabolic processes of nitrogen cycle except for anammox and the first step of nitrification. Bin 2, *Pseudomonas*, only perform ammonification and partial DNRA. Bin 3, *Acidovorax*, has capabilities of denitrification, DNRA, ammonification and the second step of nitrification. Dissimilatory arsenate reduction, arsenite oxidation, arsenite methylation, methane oxidation, methanogenesis, anammox, dissimilatory sulfate reduction, and sulfur oxidation were all absent in these bins, which is basically consistent with metagenomic and Geochip 4.0 functional analysis results (Figures 2 and 3).

**Table 1:** Overview of carbon, nitrogen, sulfur and As-metabolizing pathways in reconstructed genome bins from sample LDS.

Pathway	bin 1	bin 2	bin 3
**Carbon**			
Glycolysis	√	√	√
Citrate cycle (TCA cycle)	√	√	√
The reductive acetyl-CoA pathway	partial	partial	partial
Methanogenesis	×	×	×
Methane oxidation	×	×	×
**Nitrogen**			
Assimilatory nitrate reduction	partial	×	partial
Dissimilatory nitrate reduction	√	partial	√
Denitrification	√	×	√
Nitrogen fixation	√	×	×
Ammonification	√	√	√
Nitrification	partial	×	partial
Anammox	×	×	×
**Sulfur**			
Assimilatory sulfate reduction	√	√	√
Dissimilatory sulfate reduction	×	×	×
Sulfur oxidation	×	×	×
Sox System	partial	partial	partial
**Arsenic**			
Arsenic resistant pathway	√	√	√
Dissimilatory arsenate reduction	×	×	×
Arsenite oxidation	×	×	×
Arsenite methylation	×	×	×

Given that *Pseudomonas* dominate in microbial community and As-resistant bacteria in sample LDS (Table S5 and Figure 1), metabolic potentials of *Pseudomonas stutzeri* bin were further analyzed (Figure 3). The results show that *Pseudomonas stutzeri* could intake a variety of substrates, such as nitrate, nitrite, urea, phosphate, arsenate, sulfate, glucose and some amino acids, as well as iron, molybdate and zinc, some general cofactors of enzymes. These transporters of substrates provide a precipitate for intracellular metabolisms. The pathways of DNRA, denitrification, nitrogen fixation, ammonification, assimilatory sulfate reduction, glycolysis, citrate cycle and As-resistance are all found in the genome of *Pseudomonas stutzeri* bin. It was reported that *Pseudomonas stutzeri* is a well-known heterotrophic denitrifying bacterium, and some strains simultaneously possess the capacities of denitrification, nitrogen fixation and DNRA^64,72^. We thus propose that *Pseudomonas stutzeri* utilizes organic matter as energy and electron source to produce ammonium as well as reduce cytoplastic arsenate and pump-out arsenite to alleviate the stress of As, which leads to the accumulation of arsenite and ammonium in sample LDS. The co-existence of carbon-, nitrogen- and As-related corresponding genes in the genome of bin 2 and bin 3 supports this assumption (Table 1). In addition, a total of 11 complete genomes of *Pseudomonas stutzeri* strains retrieved from NCBI bacterial genome database also demonstrate that the As-resistant pathway is concomitant with denitrification, DNRA and ammonification (Table S7). Therefore, these ammonium-producing bacteria featuring *ars* operon may be main contributors to the transformation of arsenate to highly mobile arsenite in sample LDS.

### Mechanism of As mobilization

**Figure 4:**
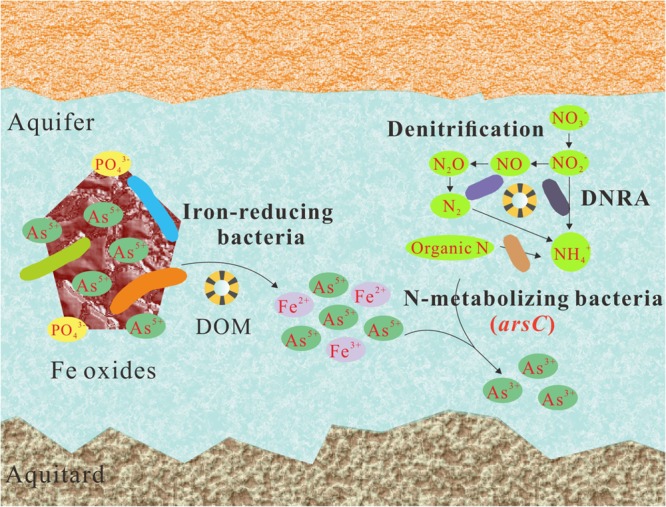
A schematic description of As mobilization mediated by microorganisms in groundwater sample LDS. OM refers to organic matter. Iron-reducing bacteria refer to *Geobacter*, *Ferribacterium* and other microorganism which are able to utilize iron as electron acceptor. N-metabolizing bacteria mainly refer to N-transforming bacteria featuring *ars* operon, such as *Pseudomonas stutzeri*.

The reductive dissolution of iron oxides is the main mechanism of As release in young groundwater systems with reducing condition^3,73^. Based on the microbial compositional and functional results from this study, we propose the pathways for arsenate release and reduction in sample LDS (Fig. 4). In this study, the iron-reducing bacteria, such as *Geobacter* and *Ferribacterium*, possibly initiate arsenate release by reductive dissolution of As-bearing iron oxides^2,13^. The competitive adsorption of phosphate may also contribute to a small fraction of dissociated arsenate^5^. The desorbed arsenate is subsequently reduced to highly mobile arsenite by some nitrogen-metabolizing bacteria featuring *ars* operon, such as *Pseudomonas stutzeri*^58,64^. Organic matter provide carbon and electron sources to fuel above biogeochemical processes^11,74^. As a consequence, high levels of ammonium, ferrous iron and arsenite were present in groundwater sample LDS (Table S1).

## Supplementary information

Supplementary Information
