# Supplementary material for: Arsenic mobilization in a high arsenic groundwater revealed by metagenomic and Geochip analyses
Source: Sci Rep. 2019 Sep 10;9:12972. doi: 10.1038/s41598-019-49365-w (PMC6736849; doi:10.1038/s41598-019-49365-w)
Supplement: Supplementary file 1 — Supplementary Information [file 41598_2019_49365_MOESM1_ESM.docx]

Supplementary Information

Arsenic mobilization in a high arsenic groundwater revealed by metagenomic and Geochip analyses

Zhou Jiang^1,2,*^, Ping Li^1,*^, Yanhong Wang^1^, Han Liu^1^, Dazhun Wei^1^, Changguo Yuan^1^, and Helin Wang^1^

^1^ State Key Laboratory of Biogeology and Environmental Geology, China University of Geosciences, Wuhan, 430074, PR China

^2^ School of Environmental Studies, China University of Geosciences, Wuhan, 430074, PR China

^*^Corresponding authors: jiangzhou@cug.edu.cn (Zhou Jiang); pli@cug.edu.cn (Ping Li)


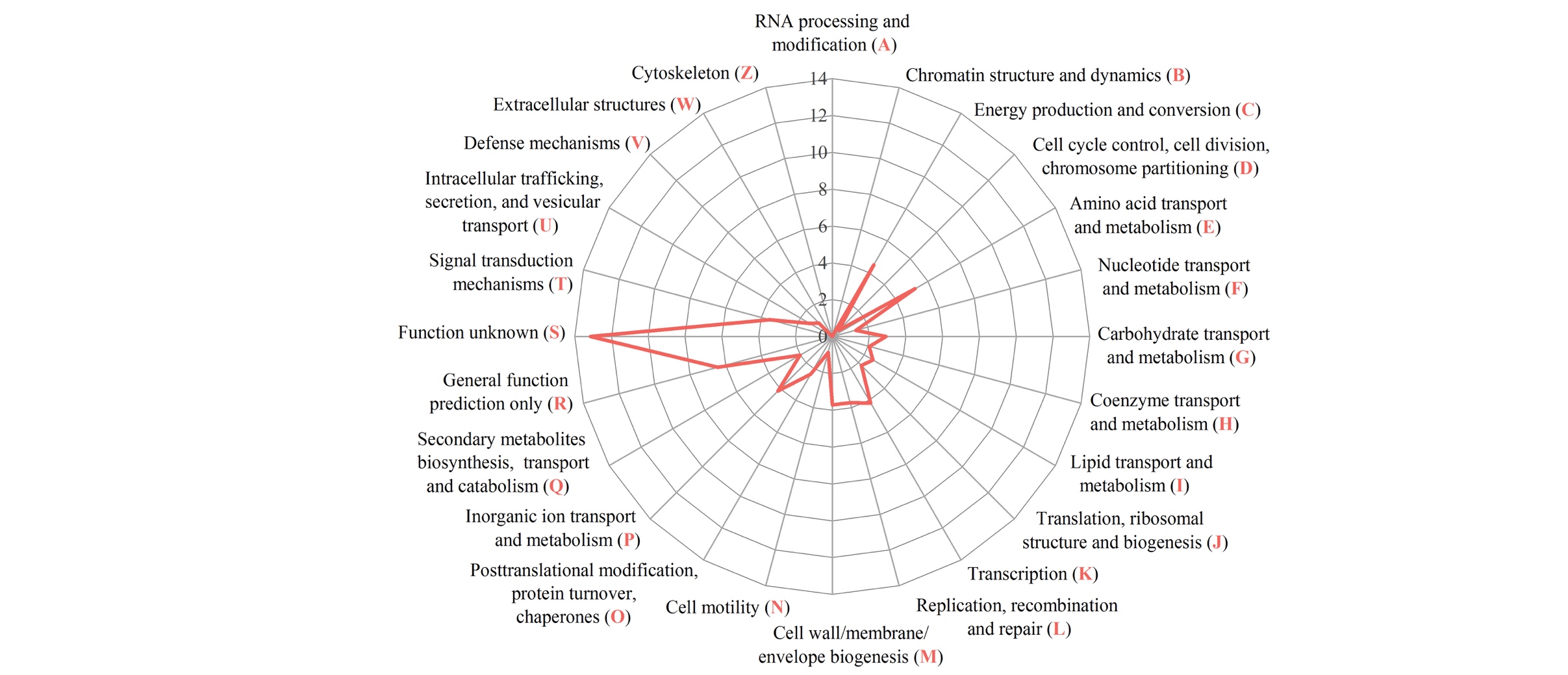


**Figure S1.** The percentage distribution of metagenomic ORFs annotated to eggNOG database in sample LDS. Letters in parentheses indicate the functional category.

| **Table S1.** Physicochemical properties of sample LDS, Hetao basin, Inner Mongolia, China | |
| --- | --- |
| Parameter | Value |
| T(°C) | 9.4 |
| pH | 8.6 |
| Electrical conductivity (µS/cm) | 1340 |
| ORP (mv) | -142 |
| Sulfide (mg/L) | 0.02 |
| Sulfate (mg/L) | bdl |
| Ammonia (mg/L) | 1.02 |
| Nitrite (mg/L) | bdl |
| Nitrate (mg/L) | 0.79 |
| Ferrous Iron (mg/L) | 0.15 |
| Total Iron (mg/L) | 0.66 |
| DOC (mg/L) | 8.58 |
| Phosphate (mg/L) | 0.24 |
| Methane (µg/L) | 204.8 |
| Arsenite (µg/L) | 991.4 |
| Arsenate (µg/L) | 288.4 |
| Monomethylarsenate, MMA (µg/L) | bdl |
| Dimethylarsonate, DMA (µg/L) | bdl |
| Total Arsenic (µg/L) | 1279.8 |
| Arsenite/Total Arsenic | 0.77 |
| bdl: below detection limit;  detection limit: Sulfide, 0.01 mg/L; Sulfate, 4.9 mg/L; Ammonia, 0.02 mg/L; Nitrite, 0.005 mg/L; Nitrate, 0.01 mg/L; Ferrous Iron, 0.03 mg/L; Total Iron, 0.03 mg/L; Phosphate, 0.07 mg/L; DOC, 1 µg/L; Methane 1 µg/L; Arsenic species, 1 µg/L | |

| Classification(%) | Ribosomal 16S DNA (N=55841) | Ribosomal 16S cDNA (N=55841) | Metagenomic Unigene annotation against NCBI-NR database (N=158405) |
| --- | --- | --- | --- |
| Phylum | | | |
| *Actinobacteria* | 0.0144 | 0.0472 | 0.0011 |
| *Bacteroidetes* | 0.1162 | 0.0319 | 0.0004 |
| *Firmicutes* | 0.0494 | 0.0157 | 0.0013 |
| *Planctomycetes* | 0.0080 | 0.0389 | 0.0001 |
| *Proteobacteria* | 0.6226 | 0.7515 | 0.9320 |
| *Verrucomicrobia* | 0.0319 | 0.0061 | 0.0001 |
| Others | 0.0741 | 0.0457 | 0.0025 |
| Unclassified | 0.0835 | 0.0628 | 0.0625 |
| Genus | | | |
| *Acidovorax* | 0.0042 | 0.0034 | 0.0860 |
| *Ferribacterium* | 0.0444 | 0.0155 | 0.0000 |
| *Flavobacterium* | 0.0234 | 0.0003 | 0.0001 |
| *Gemmata* | 0.0011 | 0.0257 | 0.0000 |
| *Geobacter* | 0.0086 | 0.0221 | 0.0001 |
| *Hydrogenophaga* | 0.0059 | 0.0037 | 0.0260 |
| *Methylobacter* | 0.0040 | 0.0200 | 0.0004 |
| *Methylocystis* | 0.0032 | 0.0257 | 0.0000 |
| *Novosphingobium* | 0.0058 | 0.0030 | 0.0224 |
| *Pseudomonas* | 0.0577 | 0.0329 | 0.5092 |
| *Sediminibacterium* | 0.0237 | 0.0066 | 0.0000 |
| *Simplicispira* | 0.0576 | 0.0668 | 0.0001 |
| *Solimonas* | 0.0321 | 0.0182 | 0.0001 |
| *Sphingobium* | 0.0094 | 0.0076 | 0.1671 |
| Others | 0.3154 | 0.3703 | 0.0917 |
| Unclassified | 0.4035 | 0.3781 | 0.0970 |

**Table S2.** Distribution of the microbial composition in groundwater LDS with abundance >2%. N in parentheses indicates the number of reads or Unigenes.

**Table S3.** General features of the metagenome data preprocessing in sample LDS

| Metagenomic assembly statistics | LDS |
| --- | --- |
| Raw reads number | 86894152 |
| Raw reads length (bp) | 13034122800 |
| Clean reads number | 76926304 |
| Clean reads length (bp) | 11387347867 |
| Clean reads Q20 (%) | 100 |
| Clean reads Q30 (%) | 97.73 |
| Clean reads GC (%) | 61 |
| Effective (%) | 87.37 |
| Scaftigs total number | 88691 |
| Scaftigs total length (bp) | 187075064 |
| Scaftigs average length (bp) | 2109 |
| Scaftigs maximum length (bp) | 912099 |
| Scaftigs N50 length (bp) | 5666 |
| Scaftigs N90 length (bp) | 693 |
| Scaftigs GC (%) | 61.05 |
| Protein-coding genes number from scaftigs (>500 bp) | 240547 |
| Protein-coding genes total length (bp) | 161430144 |
| Protein-coding genes average length (bp) | 671 |
| Protein-coding genes GC (%) | 61.84 |
| Protein-coding genes integrity number | 133065 |
| Protein-coding genes with KEGG orthologue (E value < 1E-5) | 104064 |

**Table S4.** Description of genes abbreviations in Figure 2 and Figure 3 of text body

| Abbrevation | Description | EC |
| --- | --- | --- |
| *acdA* | acetate---CoA ligase(ADP-forming) subunit alpha | 6.2.1.13 |
| *aceE* | pyruvate dehydrogenase E1 component | 1.2.4.1 |
| *aceF* | pyruvate dehydrogenase E2 component | 2.3.1.12 |
| *ackA* | acetate kinase | 2.7.2.1 |
| *acly* | ATP citrate(pro-S)-lyase | 2.3.3.8 |
| *aco* | aconitate hydrotase | 4.2.1.3 |
| *acs* | acetyl-CoA synthetase | 6.2.1.1 |
| *acsA* | anaerobic carbon-monoxide dehydrogenase catalytic subunit | 1.2.7.4 |
| *acsE* | 5-methyltetrahydrofolate corrinoid/iron sulfur protein methyltransferase | 2.1.1.258 |
| *adh1_7* | alcohol dehydrogenase 1/7 | 1.1.1.1 |
| *aio* | aerobic arsenite oxidase | - |
| *aioA* | aerobic arsenite oxidase large subunit | 1.20.2.1 1.20.9.1 |
| *aldB* | aldehyde dehydrogenase | 1.2.1.- |
| *aldh* | aldehyde dehydrogenase(NAD+) | 1.2.1.3 |
| *aldo* | fructose-bisphosphate aldolase, class I | 4.1.2.13 |
| *amo* | ammonia monooxygenase | - |
| *aprA* | adenylylsulfate reductase, subunit A | 1.8.99.2 |
| *aprB* | adenylylsulfate reductase, subunit B | 1.8.99.2 |
| *arr* | respiratory arsenate reductase | - |
| *arsA* | arsenite/tail-anchored protein-transporting ATPase | 3.6.3.16 3.6.3.- |
| *arsB* | arsenical pump membrane protein | - |
| *arsC* | arsenate reductase | 1.20.4.1 |
| *arsH* | arsenical resistance protein | - |
| *arsM* | arsenite methyltransferase | 2.1.1.137 |
| *arsR* | arsenate/arsenite/antimonite-responsive transcriptional repressor | - |
| *arx* | anaerobic arsenite oxidase | - |
| Carbon degradation | key enzymes for cellulose, chitin, lignin, hemicellulose, pectin and starch as well as other carbon compounds degradation in Geochip 4.0; KEGG orthology enzymes from glycolysis/ Gluconeogenesis (map00010) in Metagenomic | - |
| Carbon fixation | key enzymes involved six known carbon fixation pathways in Geochip 4.0; KEGG orthology enzymes from Carbon fixation pathways in prokaryotes (map00720) in Metagenomic | - |
| *cysC* | adenylylsulfate kinase | 2.7.1.25 |
| *cysD* | sulfate adenylyltransferase subunit 2 | 2.7.7.4 |
| *cysH* | phosphoadenosine phosphosulfate reductase | 1.8.4.8 1.8.4.10 |
| *cysI* | sulfite reductase (NADPH) hemoprotein beta-component | 1.8.1.2 |
| *cysJ* | sulfite reductase (NADPH) flavoprotein alpha-component | 1.8.1.2 |
| *cysK* | cysteine synthase | 2.5.1.47 |
| *cysN* | sulfate adenylyltransferase subunit 1 | 2.7.7.4 |
| *dsrA* | dissimilatory sulfite reductase alpha subunit | 1.8.99.5 |
| *dsrB* | dissimilatory sulfite reductase beta subunit | 1.8.99.5 |
| *eno* | enolase | 4.2.1.11 |
| *ethe1* | sulfur dioxygenase | 1.13.11.18 |
| *exaA* | alcohol dehydrogenase(cytochrome c) | 1.1.2.8 |
| *fbp* | fructose-1,6-bisphosphatase | 3.1.3.11 |
| *fccA* | cytochrome subunit of sulfide dehydrogenase | - |
| *fccB* | sulfide dehydrogenase(flavocytochrome c) flavoprotein chain | 1.8.2.3 |
| *fdh* | formate dehydrogenase | 1.17.1.9 |
| *fdhA* | glutathione-independent formaldehyde dehydrogenase | 1.2.1.46 |
| *fhs* | formate--tetrahydrofolate ligase | 6.3.4.3 |
| *folD* | methylenetetrahydrofolate dehydrogenase (NADP+) / methenyltetrahydrofolate cyclohydrolase | 1.5.1.5 3.5.4.9 |
| *frdA* | fumarate reductase flavoprotein subunit | 1.3.5.4 |
| *fum* | fumarate hydrotase, class I | 4.2.1.2 |
| *g6pc* | glucose-6-phosphatase | 3.1.3.9 |
| *gapA* | glyceraldehyde 3-phosphate dehydrogenase | 1.2.1.12 |
| *gapN* | glyceraldehyde-3-phosphate dehydrogenase(NADP+) | 1.2.1.9 |
| *gdh* | glutamate dehydrogenase | 1.4.1.2 1.4.1.4 |
| *glk* | glucokinase | 2.7.1.2 |
| *glpE* | thiosulfate sulfurtransferase | 2.8.1.1 |
| *glpX-SEBP* | fructose-1,6-bisphosphatase II / sedoheptulose-1,7-bisphosphatase | 3.1.3.11 3.1.3.37 |
| *cs* | citrate synthase | 2.3.3.1 |
| *gpmI* | 2,3-bisphosphoglycerate-independent phosphoglycerate mutase | 5.4.2.12 |
| *hao* | hydroxylamine dehydrogenase | 1.7.2.6 |
| *hdh* | hydrazine dehydrogenase | 1.7.2.8 |
| *hzo* | hydroxylamine oxidoreductase | - |
| *hzs* | hydrazine synthase | 1.7.2.7 |
| *icd* | isocitrate dehydrogenase | 1.1.1.42 |
| *maeA* | malate dehydrogenase (oxaloacetate-decarboxylating) | 1.1.1.38 |
| *mcrA* | methyl-coenzyme M reductase alpha subunit | 2.8.4.1 |
| *mdh1* | malate dehydrogenase | 1.1.1.37 |
| *metF* | methylenetetrahydrofolate reductase (NADPH) | 1.5.1.20 |
| *mmoX* | methane monooxygenase component A alpha chain | 1.14.13.25 |
| *mqo* | malate dehydrogenase | 1.1.5.4 |
| *napA* | periplasmic nitrate reductase | 1.7.99.- |
| *napB* | cytochrome c-type protein | - |
| *narG* | nitrate reductase, alpha subunit | 1.7.5.1 1.7.99.- |
| *narH* | nitrate reductase, beta subunit | 1.7.5.1 1.7.99.- |
| *narI* | nitrate reductase gamma subunit | 1.7.5.1 1.7.99.- |
| *nasA* | assimilatory nitrate reductase catalytic subunit | 1.7.99.- |
| *nasB* | assimilatory nitrate reductase electron transfer subunit | 1.7.99.- |
| *nifD* | nitrogenase molybdenum-iron protein alpha chain | 1.18.6.1 |
| *nifH* | nitrogenase iron protein | 1.18.6.1 |
| *nifK* | nitrogenase molybdenum-iron protein beta chain | 1.18.6.1 |
| *nirA* | ferredoxin-nitrite reductase | 1.7.7.1 |
| *nirB* | nitrite reductase (NADH) large subunit | 1.7.1.15 |
| *nirD* | nitrite reductase (NADH) small subunit | 1.7.1.15 |
| *nirK* | nitrite reductase (NO-forming) | 1.7.2.1 |
| *nirS* | nitrite reductase (NO-forming) | 1.7.2.1 1.7.99.1 |
| *norB* | nitric oxide reductase subunit B | 1.7.2.5 |
| *norC* | nitric oxide reductase subunit C | - |
| *nosZ* | nitrous-oxide reductase | 1.7.2.4 |
| *nrfA* | nitrite reductase (cytochrome c-552) | 1.7.2.2 |
| *nxrA* | nitrite oxidoreductase, alpha subunit | 1.7.5.1 1.7.99.- |
| *nxrB* | nitrite oxidoreductase, beta subunit | 1.7.5.1 1.7.99.- |
| *oforA* | 2-oxoglutarate/2-oxoacid ferredoxin oxidoreductase subunit alpha | 1.2.7.3 1.2.7.11 |
| *pckA* | phosphoenopyruvate carboxykinase(ATP) | 4.1.1.49 |
| *pfkB* | 6-phosphofructokinase 2 | 2.7.1.11 |
| *pgi* | glucose-6-phosphate isomerase | 5.3.1.9 |
| *pgk* | phosphoglycerate kinase | 2.7.2.3 |
| *pmoA* | methane monooxygenase subunit A | 1.14.18.3 1.14.99.39 |
| *ppc* | phosphoenolpyruvate carboxylase | 4.1.1.31 |
| *pps* | pyruvate, water dikinase | 2.7.9.2 |
| *prk* | phosphoribulokinase | 2.7.1.19 |
| *prps* | ribose-phosphate pyrophosphokinase | 2.7.6.1 |
| *pta* | phosphate acetyltransferase | 2.3.1.8 |
| *pyc* | pyruvate carboxylase | 6.4.1.1 |
| *pyk* | pyruvate kinase | 2.7.1.40 |
| *rbcL* | ribulose-bisphosphate carboxylase large chain | 4.1.1.39 |
| *rhdA* | thiosulfate sulfurtransferase | - |
| *rpe* | ribulose-phosphate 3-epimerase | 5.1.3.1 |
| *rpiA* | ribose 5-phosphate isomerase A | 5.3.1.6 |
| *sat* | sulfate adenylyltransferase | 2.7.7.4 |
| *sdh* | succinate dehydrogenase (ubiquinone) flavoprotein subunit | 1.3.5.1 |
| *sir* | sulfite reductase (ferredoxin) | 1.8.7.1 |
| *sor* | sulfur oxygenase/reductase | 1.13.11.55 |
| *sox* | sulfur-oxidizing protein | - |
| *soxD* | S-disulfanyl-L-cysteine oxidoreductase SoxD | 1.8.2.6 |
| *soxY* | sulfur-oxidizing protein SoxY | - |
| *sqr* | sulfide:quinone oxidoreductase | 1.8.5.4 |
| *ssuD* | alkanesulfonate monooxygenase | 1.14.14.5 |
| *sucA* | 2-Oxoglutarate dehydrogenase E1 component | 1.2.4.2 |
| *sucB* | 2-oxoglutarate dehydrogenase E2 component (dihydrolipoamide succinyltransferase) | 2.3.1.61 |
| *sucD* | succinyl-CoA synthetase alpha subunit | 6.2.1.5 |
| *suox* | sulfite oxidase | 1.8.3.1 |
| *tal* | transaldolase | 2.2.1.2 |
| *tkt* | transketolase | 2.2.1.1 |
| *tpi* | triosephosphate isomerase(TIM) | 5.3.1.1 |
| *ttrA* | tetrathionate reductase subunit A | - |
| *ureA* | urease subunit gamma | 3.5.1.5 |
| *ureB* | urease subunit beta | 3.5.1.5 |
| *ureC* | urease subunit alpha | 3.5.1.5 |
| AFU | iron(III) transport system | - |
| CYS | sulfate transport system | - |
| FHU | iron complex transport system | - |
| GTS | glucose/mannose transport system | - |
| MOD | molybdenum transport protein | - |
| NRT | nitrate/nitrite system protein | - |
| PST | phosphate transport system | - |
| SSU | sulfonate transport system | - |
| UTR | urea transport system protein | - |
| ZNU | zinc transport system | - |

EC: enzyme commission number

**Table S5**. Distribution of species harboring As,CH_4_, N, and S-metabolizing genes in sample LDS revealed by Geochip 4.0 and metagenomic sequencing. “-” refers to the species with corresponding functional gene which were not found in metagenomic sequencing data. Only species with functional gene abundance of >2% are displayed. Gene abbreviations refer to Table S4 for details.

| Geochip 4.0 | | Metagenome | | |
| --- | --- | --- | --- | --- |
| Percentage(%) | Organism | Percentage(%) | Affiliation | Similarity(%) |
| *aioA* |  |  |  |  |
| 40.0 | uncultured bacterium | - |  |  |
| 16.7 | *Burkholderia oklahomensis* C6786 | - |  |  |
| 6.8 | *Aeropyrum pernix* K1 | - |  |  |
| 4.8 | *Ochrobactrum tritici* | - |  |  |
| 4.3 | *Variovorax* sp. RM1 | - |  |  |
| 4.2 | *Chloroflexus aurantiacus* J-10-fl | - |  |  |
| 4.0 | *Hydrogenobacter* sp. GV8-4AC-C1 | - |  |  |
| 3.9 | *Pseudomonas* sp. 72 | - |  |  |
| 3.2 | *Variovorax* sp. 4-2 | - |  |  |
| 3.1 | *Alcaligenes* sp. T12RB | - |  |  |
| 2.8 | *Mesorhizobium* sp. DM1 | - |  |  |
| 2.4 | *Vibrio* sp. MED222 | - |  |  |
| *arsC* |  |  |  |  |
| 21.2 | *Delftia acidovorans* SPH-1 | 15.1 | *Pseudomonas stutzeri* DSM 4166 | 96.9 |
| 18.9 | *Maricaulis maris* MCS10 | 7.3 | *Sphingopyxis alaskensis* | 76.7 |
| 6.7 | *Aspergillus fumigatus* Af293 | 7.2 | *Sphingobium japonicum* | 78.1 |
| 5.2 | *Shewanella putrefaciens* CN-32 | 6.8 | *Sphingobium* sp. YBL2 | 87.3 |
| 4.8 | *Shewanella* sp. ANA-3 | 6.7 | *Sphingobium chlorophenolicum* | 87.9 |
| 3.7 | *Pseudomonas putida* GB-1 | 5.6 | *Pseudomonas* sp. ATCC 13867 | 86.1 |
| 2.9 | *Rhizobium leguminosarum* | 5.5 | *Pseudomonas fluorescens* Pf0-1 | 90.6 |
| 2.7 | *Rhodobacter capsulatus* | 4.6 | *Acidovorax* sp. KKS102 | 95.7 |
|  |  | 2.1 | *Sphingobium* sp. YBL2 | 89.4 |
| *arsM* |  |  |  |  |
| 38.6 | *Desulfovibrio desulfuricans subsp. desulfuricans* str. ATCC 27774 | - |  |  |
| 28.6 | *Solibacter usitatus* Ellin6076 | - |  |  |
| 11.6 | *Thermanaerovibrio acidaminovorans* DSM 6589 | - |  |  |
| 10.3 | *Pelotomaculum thermopropionicum* SI | - |  |  |
| 6.5 | *Treponema vincentii* ATCC 35580 | - |  |  |
| 4.4 | *Desulfitobacterium hafniense* Y51 | - |  |  |
| *mcrA* |  |  |  |  |
| 60.7 | uncultured archaeon | - |  |  |
| 10.1 | *Methanoculleus marisnigri* JR1 | - |  |  |
| 9.4 | *uncultured methanogenic archaeon* RC-I | - |  |  |
| 8.4 | *Methanocorpusculum labreanum* Z | - |  |  |
| 6.7 | *Methanococcus aeolicus* Nankai-3 | - |  |  |
| *pmoA or mmoX* |  |  |  |  |
| 65.8 | uncultured bacterium (*pmoA*) | - |  |  |
| 15.8 | *Azoarcus* sp. BH72 (*mmoX*) | - |  |  |
| 5.4 | *Methylomicrobium buryatense* (*mmoX*) | - |  |  |
| 4.3 | uncultured bacterium (*mmoX*) | - |  |  |
| *gdh* |  |  |  |  |
| 72.3 | *Deinococcus deserti* VCD115 | 17.7 | *Pseudomonas stutzeri* ATCC 17588 | 99.6 |
| 9.3 | *Peptoniphilus harei* | 15.8 | *Pseudomonas stutzeri* ATCC 17588 | 99.8 |
| 5.2 | *Streptococcus anginosus* | 7.9 | *Sphingobium* sp. YBL2 | 86.7 |
| 4.2 | *Bradyrhizobium* sp. ORS278 | 7.8 | *Pseudomonas fluorescens* Pf0-1 | 97.1 |
| 3.1 | *Paenibacillus* sp. JDR-2 | 6.4 | *Pseudomonas fluorescens* Pf0-1 | 96 |
| 3.0 | *Saccharopolyspora erythraea* NRRL 2338 | 5.5 | *Acidovorax* sp. KKS102 | 98.4 |
| 2.9 | *Deinococcus deserti* VCD115 | 4.6 | *Acidovorax* sp. KKS102 | 91.3 |
|  |  | 3.1 | *Sphingobium* sp. YBL2 | 74.2 |
| *ureC* |  |  |  |  |
| 21.6 | *Streptomyces ghanaensis* ATCC 14672 | 43.0 | *Pseudomonas stutzeri DSM 4166* | 100 |
| 8.1 | *Mycobacterium smegmatis* str. MC2 155 | 16.4 | *Pseudomonas fluorescens Pf0-1* | 97.9 |
| 7.4 | *Roseovarius* sp. HTCC2601 | 11.9 | *Acidovorax sp. KKS102* | 97.7 |
| 4.7 | *Saccharopolyspora erythraea* NRRL 2338 | 4.8 | *Polaromonas naphthalenivorans* | 86.5 |
| 3.6 | *Streptomyces coelicolor* A3(2) | 3.9 | *Pseudomonas fluorescens A506* | 97.7 |
| 3.2 | *Methylobacterium chloromethanicum* CM4 | 3.1 | *Sphingomonas sanxanigenens* | 85.5 |
| 3.0 | *Verminephrobacter eiseniae* EF01-2 | 2.9 | *Pseudomonas stutzeri CCUG 29243* | 93.1 |
| 2.4 | *Actinobacillus pleuropneumoniae serovar* 1 str. 4074 | 2.6 | *Pseudomonas mendocina NK-01* | 96.5 |
| 2.3 | *Prochlorococcus marinus* str. MIT 9303 | 2.5 | *Polaromonas naphthalenivorans* | 87.4 |
| 2.0 | *Bradyrhizobium* sp. BTAi1 |  |  |  |
| *nifH* |  |  |  |  |
| 58.3 | *Rhodobacter capsulatus* | 98.6 | *Pseudomonas stutzeri* A1501 | 100 |
| 14.0 | uncultured bacterium |  |  |  |
| 8.1 | uncultured nitrogen-fixing bacterium |  |  |  |
| 2.1 | unidentified nitrogen-fixing bacteria |  |  |  |
| *hao* |  |  |  |  |
| 54.6 | *Candidatus Kuenenia stuttgartiensis* | - |  |  |
| 36.3 | unidentified anaerobic bacterium | - |  |  |
| 5.4 | uncultured *planctomycete* | - |  |  |
| 3.8 | *Nitrosomonas sp. Nm143* | - |  |  |
| *napA* |  |  |  |  |
| 48.3 | uncultured bacterium | 98.0 | *Pseudomonas stutzeri* ATCC 17588 | 99.9 |
| 23.2 | *Silicibacter lacuscaerulensis* ITI-1157 |  |  |  |
| 12.5 | *Cupriavidus necator* |  |  |  |
| 6.6 | *Beggiatoa* sp. PS |  |  |  |
| 3.4 | *Sulfurospirillum barnesii* |  |  |  |
| *nrfA* |  |  |  |  |
| 31.8 | *Desulfitobacterium hafniense* DCB-2 | 100.0 | *Citrobacter freundii* | 98.7 |
| 8.3 | *Geobacter bemidjiensis* Bem |  |  |  |
| 6.0 | *Opitutus terrae* PB90-1 |  |  |  |
| 5.7 | *Geobacter* sp. M21 |  |  |  |
| 5.7 | *Bacteroides* sp. 2_1_22 |  |  |  |
| 5.5 | *Deltaproteobacterium* MLMS-1 |  |  |  |
| 4.8 | *Desulfovibrio piger* ATCC 29098 |  |  |  |
| 4.5 | *Shewanella* sp. PV-4 |  |  |  |
| 3.6 | *Shewanella loihica* PV-4 |  |  |  |
| 3.4 | *Desulfovibrio vulgaris* str. 'Miyazaki F' |  |  |  |
| 2.9 | *Desulfurivibrio alkaliphilus* AHT2 |  |  |  |
| 2.8 | *Syntrophus aciditrophicus* SB |  |  |  |
| 2.0 | *Providencia alcalifaciens* DSM 30120 |  |  |  |
| *nasA* |  |  |  |  |
| 25.8 | uncultured marine bacterium | 22.5 | *Pseudomonas stutzeri* A1501 | 99.8 |
| 22.3 | uncultured bacterium | 12.0 | *Sphingobium japonicum* | 62 |
| 20.9 | *Ralstonia solanacearum* UW551 | 11.3 | *Sphingobium japonicum* | 83.4 |
| 6.1 | *Synechococcus* sp. WH 7803 | 9.0 | *Pseudomonas fluorescens* Pf0-1 | 92.4 |
| 4.4 | uncultured prokaryote | 6.3 | *Acidovorax* sp. KKS102 | 92.5 |
| 3.1 | *Gloeothece membranacea* PCC 6501 | 5.4 | *Sphingobium chlorophenolicum* | 87.5 |
| 3.0 | *Janibacter* sp. HTCC2649 | 2.8 | *Novosphingobium aromaticivorans* | 67.7 |
| 2.9 | *Methylococcus capsulatus* str. Bath | 2.7 | *Sphingobium japonicum* | 55.9 |
| 2.4 | *Rhodobacter capsulatus* | 2.6 | *Sphingobium japonicum* | 84.8 |
|  |  | 2.5 | *Acidovorax* sp. KKS102 | 75 |
|  |  | 2.2 | *Pseudomonas fluorescens* UK4 | 93.1 |
|  |  | 2.1 | *Sphingobium chlorophenolicum* | 84.6 |
|  |  | 2.1 | *Sphingobium chlorophenolicum* | 85.2 |
|  |  | 2.0 | *Sphingobium* sp. YBL2 | 57.3 |
| *nirA* |  |  |  |  |
| 25.1 | *Gloeobacter violaceus* PCC 7421 | - |  |  |
| 20.6 | *Microcystis aeruginosa* NIES-843 | - |  |  |
| 13.3 | *Opitutus terrae* PB90-1 | - |  |  |
| 8.7 | *Haloquadratum walsbyi* DSM 16790 | - |  |  |
| 7.4 | *Synechococcus* sp. WH 7803 | - |  |  |
| 6.7 | *Microcystis aeruginosa* NIES-843 | - |  |  |
| 4.3 | *Rhodococcus jostii* RHA1 | - |  |  |
| 3.9 | *Synechococcus sp.* JA-2-3B'a(2-13) | - |  |  |
| 2.8 | *Archaeoglobus fulgidus* DSM 4304 | - |  |  |
| 2.5 | *Acaryochloris marina* MBIC11017 | - |  |  |
| 2.4 | *Thermosynechococcus elongatus* BP-1 | - |  |  |
| 2.3 | *Nostoc* sp. PCC 7120 | - |  |  |
| *nirB* |  |  |  |  |
| 35.2 | *Streptomyces avermitilis* MA-4680 | 21.4 | *Pseudomonas stutzeri* A1501 | 98.9 |
| 26.0 | *Rothia mucilaginosa* ATCC 25296 | 19.6 | *Pseudomonas stutzeri* ATCC 17588 | 99.6 |
| 12.8 | *Hyphomonas neptunium* ATCC 15444 | 9.3 | *Sphingobium chlorophenolicum* | 90.5 |
| 11.8 | *Saccharopolyspora erythraea* NRRL 2338 | 8.2 | *Pseudomonas fluorescens* Pf0-1 | 98.9 |
| 6.9 | *Verrucomicrobiae* bacterium V4 | 6.0 | *Acidovorax* sp. KKS102 | 96.7 |
| 5.6 | *Roseobacter denitrificans* OCh 114 | 6.0 | *Variovorax paradoxus* EPS | 85.1 |
|  |  | 2.3 | *Acidovorax avenae* | 85.7 |
|  |  | 2.2 | *Sphingobium chlorophenolicum* | 89.7 |
|  |  | 2.2 | *Novosphingobium aromaticivorans* | 88.2 |
| *narG* |  |  |  |  |
| 77.6 | uncultured bacterium | 66.6 | *Pseudomonas stutzeri* A1501 | 99.8 |
| 19.8 | unidentified bacterium | 18.1 | *Acidovorax* sp. KKS102 | 95.9 |
|  |  | 5.2 | *Methylibium petroleiphilum* | 89.5 |
|  |  | 3.5 | *Pseudomonas fluorescens* F113 | 100 |
| *nirK* |  |  |  |  |
| 90.1 | uncultured bacterium | 64.0 | *Pseudomonas* sp. ATCC 13867 | 83.1 |
| 3.8 | *Agrobacterium tumefaciens* str. C58 | 18.9 | *Massilia* sp. NR 4-1 | 69.3 |
| 3.5 | *Haloferax denitrificans* | 17.1 | *Pseudomonas stutzeri* RCH2 | 97.7 |
| *nirS* |  |  |  |  |
| 72.2 | uncultured bacterium | 88.9 | *Pseudomonas stutzeri* A1501 | 100 |
| 12.9 | uncultured organism | 7.4 | *Pseudomonas* sp. TKP | 89.5 |
| 7.2 | *Paracoccus pantotrophus* |  |  |  |
| 2.4 | *Hydrogenobacter thermophilus* |  |  |  |
| 2.2 | uncultured temperate forest soil bacterium |  |  |  |
| *norB* |  |  |  |  |
| 64.5 | uncultured bacterium | 56.7 | *Pseudomonas stutzeri* DSM 4166 | 100 |
| 13.4 | *Sinorhizobium meliloti* 1021 | 19.5 | *Acidovorax* sp. KKS102 | 93.7 |
| 6.3 | *Nitrosomonas marina* | 4.8 | *Sphingomonas wittichii* | 100 |
| 3.1 | *Agrobacterium tumefaciens* str. C58 | 4.3 | *Xanthobacter autotrophicus* | 94.8 |
| 2.8 | *Roseobacter denitrificans* | 4.1 | *Pseudomonas* sp. TKP | 96 |
| 2.3 | *Hahella chejuensis* KCTC 2396 | 2.2 | *Xanthobacter autotrophicus* | 88.1 |
| *nosZ* |  |  |  |  |
| 47.6 | uncultured temperate forest soil bacterium CZ1441 | 69.8 | *Pseudomonas stutzeri* A1501 | 100 |
| 27.5 | uncultured bacterium | 17.4 | *Alicycliphilus denitrificans* BC | 86.8 |
| 19.2 | unidentified bacterium | 5.1 | *Pseudomonas* sp. TKP | 95.1 |
| 2.4 | *Thiobacillus denitrificans* ATCC 25259 | 5.0 | *Pseudomonas* sp. TKP | 92.9 |
| *hzo* |  |  |  |  |
| 95.8 | uncultured *planctomycete* | - |  |  |
| 3.0 | *Candidatus Brocadia* sp. enrichment culture clone HZO4 | - |  |  |
| *aprA* |  |  |  |  |
| 32.8 | *Archaeoglobus profundus* DSM 5631 | - |  |  |
| 28.4 | uncultured bacterium | - |  |  |
| 13.2 | *Desulfovibrio ferrophilus* | - |  |  |
| 10.0 | *Robbea* sp. 2 SB-2008 associated bacterium | - |  |  |
| 7.7 | endosymbiont of *Inanidrilus exumae* | - |  |  |
| 6.1 | uncultured *gammaproteobacterium* | - |  |  |
| *cysI* |  |  |  |  |
| 12.4 | *Streptomyces cf. griseus* XylebKG-1 | 26.2 | *Pseudomonas stutzeri* DSM 4166 | 99.6 |
| 10.9 | *Cardiobacterium hominis* ATCC 15826 | 13.4 | *Sphingobium* sp. YBL2 | 89.7 |
| 9.2 | *Neisseria flavescens* SK114 | 10.2 | *Pseudomonas mandelii* | 97.6 |
| 6.7 | *Stigmatella aurantiaca* DW4/3-1 | 9.4 | *Pseudomonas fluorescens* Pf0-1 | 94.1 |
| 6.5 | *Streptomyces roseosporus* NRRL 11379 | 7.1 | *Verminephrobacter eiseniae* | 85.2 |
| 6.4 | *Nocardia farcinica* IFM 10152 | 2.8 | *Polaromonas* sp. JS666 | 77 |
| 5.0 | *Pseudomonas fluorescens* SBW25 | 2.7 | *Novosphingobium aromaticivorans* | 95.6 |
| 4.8 | *Bradyrhizobium japonicum* USDA 110 | 2.5 | *Sphingobium japonicum* | 92.3 |
| 3.6 | *Dickeya dadantii* 3937 | 2.4 | *Pseudomonas poae* | 98.7 |
| 2.4 | *Pseudomonas fluorescens* WH6 | 2.3 | *Pseudomonas fluorescens* UK4 | 96.2 |
|  |  | 2.2 | *Acinetobacter pittii* | 91.4 |
| *sir* |  |  |  |  |
| 34.7 | *Neisseria elongata* subsp. glycolytica ATCC 29315 | - |  |  |
| 8.5 | *Cylindrospermopsis raciborskii* CS-505 | - |  |  |
| 7.5 | *Tuber melanosporum* Mel28 | - |  |  |
| 5.4 | *Aspergillus nidulans* FGSC A4 | - |  |  |
| 4.5 | *Crocosphaera watsonii* WH 8501 | - |  |  |
| 3.8 | *Truepera radiovictrix* DSM 17093 | - |  |  |
| 3.5 | *Yersinia enterocolitica* subsp. palearctica 105.5R(r) | - |  |  |
| 3.1 | *Synechococcus* sp. WH 5701 | - |  |  |
| 2.4 | *Granulibacter bethesdensis* CGDNIH1 | - |  |  |
| 2.1 | *Cyanothece* sp. PCC 7425 | - |  |  |
| 2.1 | *Lachancea thermotolerans* | - |  |  |
| 2.1 | *Nitrosococcus oceani* ATCC 19707 | - |  |  |
| *dsrA* |  |  |  |  |
| 47.5 | *Pyrobaculum calidifontis* JCM 11548 | 100.0 | *Sulfuritalea hydrogenivorans* | 74.3 |
| 35.1 | uncultured sulfate-reducing bacterium |  |  |  |
| 4.0 | uncultured sulfate-reducing bacterium UMTRAdsr857-10 |  |  |  |
| 3.0 | uncultured bacterium |  |  |  |
| *dsrB* |  |  |  |  |
| 66.0 | uncultured sulfate-reducing bacterium | 100.0 | *Burkholderiales* bacterium GJ-E10 | 87.4 |
| 8.3 | uncultured bacterium |  |  |  |
| 6.5 | *Magnetococcus* sp. MC-1 |  |  |  |
| 3.9 | *Desulfospira joergensenii* |  |  |  |
| 2.2 | *Syntrophobacter fumaroxidans* |  |  |  |
| *sqr* |  |  |  |  |
| 77.9 | *Acaryochloris marina* MBIC11017 | 23.1 | *Pseudomonas stutzeri* A1501 | 98.8 |
| 12.8 | *Rhodospirillum centenum* SW | 12.5 | *Sphingopyxis fribergensis* | 69.7 |
| 6.5 | *Allochromatium vinosum* DSM 180 | 12.1 | *Sphingopyxis fribergensis* | 74.9 |
| 2.8 | *Rhodobacter capsulatus* SB 1003 | 9.3 | *Pseudomonas fluorescens* Pf0-1 | 88.6 |
|  |  | 2.9 | *Sphingopyxis fribergensis* | 71.1 |
|  |  | 2.6 | *Sphingomonas wittichii* | 88.4 |
|  |  | 2.5 | *Pseudomonas fluorescens* UK4 | 100 |
| *fccAB* |  |  |  |  |
| 59.5 | *Rhodobacter sphaeroides* ATCC 17025 | - |  |  |
| 13.7 | *alphaproteobacterium* BAL199 | - |  |  |
| 3.3 | *Methylobacillus flagellatus* KT | - |  |  |
| 2.1 | *Thiomonas* sp. 3As | - |  |  |
| *sox* |  |  |  |  |
| 38.3 | *Prosthecochloris vibrioformis* DSM 265 | 24.3 | *Pseudomonas stutzeri* ATCC 17588 | 98.8 |
| 7.4 | uncultured bacterium | 13.4 | *Sphingobium* sp. YBL2 | 71.1 |
| 6.8 | *Bradyrhizobium* sp. ORS278 | 3.5 | *Sphingobium* sp. YBL2 | 69.5 |
| 5.5 | *Nitrobacter hamburgensis* X14 | 3.4 | *Janthinobacterium* sp. Marseille | 73.2 |
| 5.3 | *Ruegeria* sp. R11 | 3.1 | *Sulfuritalea hydrogenivorans* | 74.3 |
| 4.3 | *Methylobacterium extorquens* AM1 | 3.0 | *Comamonas thiooxydans* | 68.3 |
| 3.5 | *Methylobacterium populi* BJ001 | 2.9 | *Anaeromyxobacter dehalogenans* 2CP-C | 66 |
| 2.9 | *Leucothrix mucor* | 2.8 | *Leptothrix cholodnii* | 76.6 |
| 2.1 | *Allochromatium vinosum* DSM 180 | 2.8 | *Serpentinomonas raichei* | 58.3 |

**Table S6.** Overview of genomic bins reconstructed from metagenomic in sample LDS

| Genome bins | bin 1 | bin 2 | bin 3 |
| --- | --- | --- | --- |
| Genome size, bp | 4489785 | 5483160 | 5275946 |
| Number of scaffolds | 36 | 36 | 61 |
| Mean scafford lengh, bp | 124716 | 152310 | 86491 |
| N50 value, bp | 186746 | 234428 | 161804 |
| No. of protein coding genes | 4238 | 5084 | 5092 |
| No. of rRNA | 0 | 0 | 1 |
| No. of tRNA | 49 | 49 | 42 |
| Coding density, % | 94 | 93 | 97 |
| Completeness, % | 99.28 | 96.47 | 98.86 |
| Contamination, % | 0.14 | 0.77 | 1.1 |
| Strain heterogeneity, % | 0 | 0 | 0 |
| GC content, % | 64.34 | 59.29 | 64.65 |
| lineage | *Pseudomonas stutzeri* | *Pseudomonas* | *Acidovorax* |

**Table S7.** Description of nitrogen- and arsenic-metabolizing processes in 11 complete genomes of *Pseudomonas stutzeri* strains retrieved from bacterial genome database in NCBI in Oct 7, 2018

| Strain | ANRN | DNRA | Denitrification | Nitrogen fixation | Ammonification | Nitrification | Anammox | Arsenic resistant pathway |
| --- | --- | --- | --- | --- | --- | --- | --- | --- |
| 28a24 | × | √ | × | × | √ | × | × | √ |
| CGMCC 1.1803 | × | √ | √ | × | √ | × | × | √ |
| A1501 | × | √ | √ | √ | √ | × | × | √ |
| DSM 4166 | × | √ | √ | √ | √ | × | × | √ |
| CCUG 29243 | × | √ | √ | × | √ | × | × | √ |
| DSM 10701 | × | √ | √ | × | √ | × | × | √ |
| RCH2 | × | √ | √ | × | √ | × | × | √ |
| 19SMN4 | × | √ | √ | × | √ | × | × | √ |
| SLG510A3-8 | × | √ | √ | × | √ | × | × | √ |
| 273 | × | √ | √ | × | √ | × | × | √ |
| DW2-1 | × | √ | √ | × | √ | × | × | √ |
| 1W1-1A | × | √ | √ | × | √ | × | × | √ |
| SGAir0442 | × | √ | √ | √ | √ | × | × | √ |
| ANRN: Assimilatory nitrate reduction to ammonia; DNRA: Dissimilatory nitrate reduction to ammonia | | | | | | | | |
